# The Impact of Vitiligo on Patients' Quality of Life: Retrospective Observational Study

**DOI:** 10.1111/ijd.17998

**Published:** 2025-07-31

**Authors:** Vincenzo Picone, Luigi Coronella, M. Turco, Marianna Cimmino, Cataldo Patruno, Massimiliano Scalvenzi, Maddalena Napolitano

**Affiliations:** ^1^ Section of Dermatology, Department of Clinical Medicine and Surgery University of Naples Federico II Naples Italy; ^2^ Department of Medicine and Health Sciences Vincenzo Tiberio University of Molise Campobasso Italy

**Keywords:** biologics, JAK, quality of life, vitiligo

Vitiligo is a chronic autoimmune skin condition marked by the progressive destruction of melanocytes, resulting in depigmented patches [1]. While its pathogenesis involves genetic, environmental, and immunological factors, the psychosocial burden remains significant and often under‐recognized [2]. This study explored the psychological and emotional impact of non‐segmental vitiligo (NSV) on patients' quality of life (QoL), as well as the short‐term effects of topical ruxolitinib treatment, a new emerging therapies [3]. A total of 158 Italian patients over the age of 12 were enrolled, and questionnaires assessing QoL, disease severity, and emotional well‐being were administered both at baseline and, for 91 of them, after 4 weeks of treatment with ruxolitinib cream applied twice daily. At baseline, the psychological burden of vitiligo appeared substantial. Sociodemographic data are summarized in Table 1. The Dermatology Life Quality Index (DLQI) showed a high mean score of 22.4 ± 7.2 standard deviation (SD), reflecting a strong impact of the disease on daily life. Anxiety and depression were also prevalent, with mean Hospital Anxiety and Depression Scale‐Anxiety (HADS‐A) and Depression (HADS‐D) scores of 15.3 ± 4.9 SD and 16.8 ± 4.2 SD, respectively. The Patient Global Impression of Severity (PGI‐S) average score was 2.8 ± 0.5 SD, while the Vitiligo Impact Treatment Score (VITs), evaluating treatment perception and satisfaction, was 37.3 ± 12.5 SD. These results collectively indicate that vitiligo significantly interferes with patients' psychological and emotional well‐being, as well as with their daily functioning. After 4 weeks of ruxolitinib treatment, a significant improvement was observed in all the QoL‐related indicators (Figure 1). DLQI decreased markedly to 10.1 ± 8.7 SD, indicating a reduced negative impact on life. Similarly, the PGI‐S dropped to 1.5 ± 0.7 SD, and the HADS scores also showed meaningful reductions: HADS‐A to 7.3 ± 6.2 SD and HADS‐D to 8.1 ± 6.8 SD. The VITs score lowered to 21.5 ± 16.6 SD, suggesting greater satisfaction with the treatment and better perceived efficacy. All these improvements were statistically significant (*p* < 0.05), underscoring the positive psychological impact of ruxolitinib cream even after a relatively short period. However, the same trend was not observed for clinical improvement. The Facial Vitiligo Area Scoring Index (F‐VASI) and Total VASI (T‐VASI) scores, used to evaluate physical extent and severity of depigmentation, showed only minimal, non‐significant reductions (F‐VASI from 7.5 ± 2. SD 1 to 7.1 ± 1.9 SD, and T‐VASI from 23.2 ± 5.6 SD to 22.3 ± 5.4 SD, *p* > 0.05). These findings imply that although visible repigmentation might not be substantial after just 4 weeks, patients still perceive considerable improvement in their overall well‐being. A particularly important aspect highlighted by the study was the correlation between disease duration and perceived QoL impact. Patients who had lived with vitiligo for less than a year reported the highest DLQI scores (mean 24.8 ± 5.8 SD), indicating that the initial phase after diagnosis is the most emotionally and socially challenging. In contrast, those with 1–5 years of disease had a mean DLQI of 21.3 ± 7.1 SD, and those with more than 5 years reported the lowest (18.7 ± 7.4 SD), suggesting some degree of psychological adaptation over time. Interestingly, the VITs scores showed the opposite trend. Patients with a more recent onset of vitiligo had lower VITs scores (30.1 ± 10.2 SD), implying more motivation and greater compliance with therapy. Conversely, those with long‐standing vitiligo had higher VITs scores (up to 43.2 ± 11.8 SD), potentially reflecting therapeutic fatigue and dissatisfaction with previous treatments. These results support the theory that early psychological support and timely therapeutic intervention are crucial, especially shortly after diagnosis when the psychological toll is highest [4]. The apparent improvement in QoL despite minimal clinical changes may be linked to the “expectation effect,” where patient optimism and belief in new therapies enhance perceived outcomes and emotional well‐being. In summary, this study demonstrates that NSV significantly impairs QoL, particularly in the early stages, and that ruxolitinib cream can offer meaningful psychological benefits within just 4 weeks of use, even before visible skin improvement occurs [5]. This emphasizes the importance of a holistic treatment approach that prioritizes not just physical symptoms but also mental health and emotional resilience in managing vitiligo.

**TABLE 1:** Sociodemographic and clinical data.

Number of patients	158
Number of men (%)	61 (38.6%)
Number of women (%)	97 (61.4%)
Average age (in years) ± SD	40 ± 9.1
Average age at vitiligo onset ± SD	26 ± 7.5
Positive familiarity for vitiligo; number (%)	21 (13.3%)
Average duration of disease; number (%)	
< 1 year	32 (20.3%)
Between 1 and 5 years	58 (36.7%)
> 5 years	68 (43%)
Areas of cutaneous involvement; number (%)	
Face	139 (88%)
Hands	107 (67.7%)
Feet	38 (24%)
Trunk	46 (29.1%)
Genitals	67 (42.4%)
Comorbidities, number (%)	
Autoimmune thyroiditis	55 (34.1%)
Inflammatory bowel diseases (IBD)	21 (13.3%)
Type 1 mellitus diabetes	5 (3.2%)
Celiac disease	5 (3.2%)
Previous treatments; number (%)	
Narrow‐Band UVB (311 nm)	116 (73.4%)
Topical corticosteroids	124 (78.5%)
Topical calcineurin inhibitors	63 (39.9%)

Abbreviation: SD, standard deviation.

**FIGURE 1:**
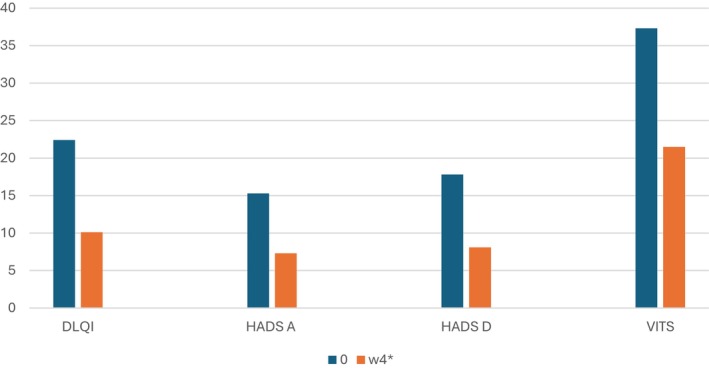
Mean scores of Qol questionnaires at baseline and after 4 weeks of treatment with ruxolitinib cream. Improvement was observed in all the QoL‐related indicators (*p* < 0.05)*.

## Consent

The patients in this manuscript have given written informed consent to publication of their case details.

## Conflicts of Interest

M.N. acted as speaker, consultant, and advisory board member for Sanofi, AbbVie, Lilly, Leo Pharma, and Pfizer; C.P. acted as investigator, speaker, consultant, and advisory board member for AbbVie, Eli Lilly, Novartis, Pfizer, Pierre Fabre, and Sanofi. The other authors declare no conflicts of interest.

## Data Availability

The data that support the findings of this study are available from the corresponding author upon reasonable request.
